# Real-World Evaluation of Disease Progression After CDK 4/6 Inhibitor Therapy in Patients With Hormone Receptor-Positive Metastatic Breast Cancer

**DOI:** 10.1093/oncolo/oyad035

**Published:** 2023-03-22

**Authors:** Malinda T West, Shaun M Goodyear, Evthokia A Hobbs, Andy Kaempf, Thomas Kartika, Jessica Ribkoff, Brie Chun, Zahi I Mitri

**Affiliations:** OHSU Knight Cancer Institute, Oregon Health & Science University, Portland, OR, USA; University of Wisconsin Carbone Cancer Center, Madison, WI, USA; OHSU Knight Cancer Institute, Oregon Health & Science University, Portland, OR, USA; OHSU Knight Cancer Institute, Oregon Health & Science University, Portland, OR, USA; OHSU Knight Cancer Institute, Oregon Health & Science University, Portland, OR, USA; OHSU Knight Cancer Institute, Oregon Health & Science University, Portland, OR, USA; Internal Medicine Residency Program, Providence Portland Medical Center, Portland, OR, USA; OHSU Knight Cancer Institute, Oregon Health & Science University, Portland, OR, USA; Internal Medicine Residency Program, Providence Portland Medical Center, Portland, OR, USA; OHSU Knight Cancer Institute, Oregon Health & Science University, Portland, OR, USA; British Columbia Cancer Agency, Vancouver, CA, USA

**Keywords:** CDK 4/6 inhibitor resistance, hormone receptor positive metastatic breast cancer, PTEN mutation

## Abstract

**Background:**

Cyclin-dependent kinase 4/6 inhibitors (CDKi) have changed the landscape for treatment of patients with hormone receptor positive, human epidermal growth factor receptor 2-negative (HR+/HER−) metastatic breast cancer (MBC). However, next-line treatment strategies after CDKi progression are not yet optimized. We report here the impact of clinical and genomic factors on post-CDKi outcomes in a single institution cohort of HR+/HER2− patients with MBC.

**Methods:**

We retrospectively reviewed the medical records of patients with HR+/HER2− MBC that received a CDKi between April 1, 2014 and December 1, 2019 at our institution. Data were summarized using descriptive statistics, the Kaplan-Meier method, and regression models.

**Results:**

We identified 140 patients with HR+/HER2− MBC that received a CDKi. Eighty percent of patients discontinued treatment due to disease progression, with a median progression-free survival (PFS) of 6.0 months (95% CI, 5.0-7.1), whereas those that discontinued CDKi for other reasons had a PFS of 11.3 months (95% CI, 4.6-19.4) (hazard ratio (HR) 2.53, 95% CI, 1.50-4.26 [*P* = .001]). The 6-month cumulative incidence of post-CDKi progression or death was 51% for the 112 patients who progressed on CDKi. Patients harboring *PTEN* mutations pre-CDKi treatment had poorer clinical outcomes compared to those with wild-type *PTEN*.

**Conclusion:**

This study highlights post-CDKi outcomes and the need for further molecular characterization and novel therapies to improve treatments for patients with HR+/HER2− MBC.

## Teaser

Next-line treatment strategies for patients with HR+/HER2– metastatic breast cancer (MBC) after CDKi progression are not yet optimized. This article reports on the effect of clinical and genomic factors on post-CDKi outcomes in a single institution cohort of HR+/HER2– patients with MBC.

**Implications for Practice**

The unfortunate expectation of cyclin-dependent kinase 4/6 inhibitor therapy resistance and disease progression continues to challenge the treatment and management of patients with metastatic hormone receptor-positive human epidermal growth factor 2 receptor-negative breast cancers. In this single-center, retrospective review, real-world clinicopathological features, and mutational profiling points to a subset of progressing tumors that are more aggressive upon discontinuation of cyclin-dependent kinase 4/6 inhibitor therapy. This article underscores the clinical challenge with deciding upon optimal salvage strategy for patients experiencing disease progression on cyclin-dependent kinase 4/6 inhibitor therapy.

## Introduction

Endocrine therapy (ET) is a cornerstone treatment for patients with hormone receptor-positive (HR+), human epidermal growth factor receptor 2-negative (HER2−) breast cancer, a subtype that accounts for two-thirds of all advanced or metastatic breast cancers (MBC).^1^ Treatment of MBC with ET is often augmented with targeted agents, including the small molecule inhibitors of cyclin-dependent kinase 4/6 (CDKi).^2^ The addition of CDKi (eg, palbociclib, ribociclib, and abemaciclib) to the treatment landscape have proven clinically beneficial. Compared to ET alone, the addition of CDKi nearly doubles the median progression-free survival (PFS), from 14-16 to 25-28 months,^3-8^ and improves overall survival by 7-12 months.^9-12^ Despite these clinical trial results, tumors develop resistance to CDKi that requires switching to different endocrine, targeted, or chemotherapy approaches for the treatment of HR+/HER2− MBC.^13-19^ Emerging evidence shows CDKi therapy may promote new molecular alterations in tumor cells that could limit the effectiveness of subsequent currently approved regimens.^18,20-26^ In addition, over-activation of the phosphoinositide-(3)-kinase (PI3K) signaling pathway is common in HR+/HER2− MBC and may contribute to endocrine and CDKi resistance. PI3K inhibitors have shown activity in PI3K-mutated tumors post-CDKi therapy; however, it is unknown if targeting this pathway can prevent CDKi resistance from developing. The need to optimize post-CDKi therapies is underscored by reports of rapid disease progression after CDKi discontinuation (referred herein as post-CDKi). Thus, there is great need to understand clinical and tumor-specific characteristics to better guide post-CDKi therapy selection, particularly as the field lacks clinical practice guidance for next-line strategies.

Here we present findings of a single-institution retrospective review evaluating post-CDKi outcomes of patients with HR+/HER2− MBC. The study objectives were to describe patient and disease characteristics from the time of metastatic diagnosis, estimate rates of disease progression, or death following CDKi discontinuation, and determine clinical and molecular predictors of tumor progression.

## Methods

### Study Population

We performed a retrospective review of patient medical records from the Oregon Health & Science University’s Knight Cancer Institute (Portland, Oregon, USA) for patients over 18 years of age, with HR+/HER2− MBC, who were initially prescribed a CDKi between April 1, 2014 and December 1, 2019. We excluded patients whose reason for CDKi discontinuation was death or transition to hospice (as they lacked post-CDKi follow-up data), male patients, and those who failed to receive one cycle of a CDKi.

Patient information was collected from the electronic health records database, including patient demographics, tumor characteristics, location of metastatic disease, presence of visceral (ie, liver, lung, ascites, pleural effusion, and metastases in the central nervous system) or non-visceral (ie, bone, skin, and lymph node) metastatic lesions, all treatments in the metastatic setting prior to CDKi including ET and chemotherapy, duration of CDKi, accompanying ET, reason for CDKi discontinuation, and type and duration of post-CDKi treatment. Additionally, next-generation sequencing (NGS) findings on pre-CDKi samples were analyzed for patients with these results available.

The study was approved by the OHSU Institutional Review Board and granted a waiver of informed consent due to its retrospective nature (OHSU IRB#19888).

### Statistical Analysis

Descriptive analyses were performed to characterize patient demographic, disease, and treatment variables pertaining to the time of metastatic diagnosis onwards. Categorical variables were represented with frequency counts and percentages while continuous variables were summarized by the median, interquartile range [IQR], and range. The primary outcome of interest was post-CDKi PFS and secondary outcome was post-CDKi OS, each measured from the date of CDKi discontinuation. Disease progression was determined by physician documentation of clinical and/or radiographic findings, such as patient symptoms, physical exam findings, increase in disease burden on imaging, and start of a new therapy. As RECIST criteria was not consistently reported for our retrospective cohort, we did not analyze overall response rate or clinical benefit rate. Follow-up time was estimated with the reverse Kaplan-Meier method. The standard Kaplan-Meier method was used to estimate survival distributions and, in the absence of competing risks, cumulative incidence was computed as 1-minus the Kaplan-Meier survival estimate. The log-rank test was applied to compare survival distributions in the absence of covariates. Cox proportional hazards regression models were fit to quantify and test the association between candidate predictor variables and time-to-event outcomes. All considered predictors were determined at or before the survival start time (ie, CDKi discontinuation) except for post-CDKi therapy, which was defined as a time-varying categorical predictor and evaluated with the extended Kaplan-Meier estimator and a Cox regression model that re-evaluated patient therapy at each observed event time. Rapid disease progression (ie, the occurrence of disease progression or death within 6 months after a patient discontinued a CDKi regimen) was modeled by logistic regression after removing patients (4%) censored before the 6-month threshold. Multivariable model selection for each study outcome, performed on the full patient cohort as well as the subset of patients with available pre-CDKi NGS results, considered all predictors with univariable model Wald test *P*-values < .20 and consisted of backward elimination with an Akaike’s Information Criterion (AIC) stopping rule. *P*-values < .05 were considered statistically significant and there was no adjustment for multiple comparisons. Statistical analyses were conducted with R version 4.2.1.

## Results

### Patient Cohort

We identified 140 HR+/HER2− MBC patients that received a CDKi at our institution between 2014 and 2019 and discontinued CDKi for any reason other than death or transition to hospice. Patient and clinicopathological data are summarized in Table 1. Nearly all patients were post-menopausal with a median age of 65 (range 34-87) at the time of CDKi discontinuation. The majority (76%) had metastatic disease arising from earlier stage disease, with visceral disease present in 51% of patients at the time of metastatic diagnosis. Two-thirds of patients received treatments in the metastatic setting prior to CDKi and the median time from metastatic diagnosis to start of CDKi was 7.4 months (IQR 1.4-31.2 months). Palbociclib was the predominant CDKi given (93%). Letrozole (52%) and fulvestrant (40%) were the most common ETs given in combination with a CDKi. The median duration of CDKi therapy was 8.7 months (IQR 3.5-17.4 months), with progression being the primary reason for discontinuation (112/140, 80%). Treatments after discontinuing CDKi included chemotherapy (44%), ET (25%), targeted therapy (including everolimus and alpelisib, 21%), no further treatment (7%), or subsequent CDKi (3%).

**Table 1.** Clinicopathological characteristics of study cohort.

Clinical characteristic	Summary statistics *N* (%) or median (IQR)
Number of patients	140
Age at CDKi discontinuation	
<65 years	72 (51%)
≥65 years	68 (49%)
De novo metastatic breast cancer	
No	106 (76%)
Yes	34 (24%)
Metastasis type (at time of metastatic diagnosis)	
Visceral^a^	71 (51%)
Non-visceral	69 (49%)
Pre-CDKi therapy in metastatic setting	
No	47 (34%)
Yes	93 (66%)
Pre-CDKi chemo in metastatic setting	
No	100 (71%)
Yes^b^	40 (29%)
Pre-CDKi anastrozole in met. setting	
No	118 (84%)
Yes	22 (16%)
Pre-CDKi letrozole in met. setting	
No	88 (63%)
Yes	52 (37%)
Pre-CDKi exemestane in met. setting	
No	111 (79%)
Yes	29 (21%)
Pre-CDKi fulvestrant in met. setting	
No	99 (71%)
Yes	41 (29%)
Pre-CDKi tamoxifen in met. setting	
No	126 (90%)
Yes	14 (10%)
Pre-CDKi everolimus in met. setting	
No	126 (90%)
Yes	14 (10%)
Pre-CDKi capecitabine in met. setting	
No	111 (79%)
Yes	29 (21%)
Time between metastatic diagnosis and start of CDKi therapy	Median (IQR): 7.4 months (1.4-31.2)
Type of CDKi (first regimen)	
Palbociclib	130 (93%)
Ribociclib	3 (2%)
Abemaciclib	7 (5%)
Endocrine therapy (specific) during CDKi^c^	
Letrozole	73 (52%)
Fulvestrant	56 (40%)
Anastrozole	9 (6%)
Tamoxifen	1 (1%)
None	1 (1%)
Exemestane	0 (0%)
Endocrine therapy (group) during CDKi^d^	
No fulvestrant	84 (60%)
Fulvestrant	56 (40%)
Duration of CDKi^c^	Median (IQR): 8.7 months (3.5-17.4)
<12 months	81 (65%)
≥12 months	49 (35%)
Reason for CDKi discontinuation (specific)	
Progression	112 (80%)
Adverse event	22 (16%)
Surgery or other therapy	2 (1%)
Rising tumor marker	3 (2%)
Patient preference	1 (1%)
Reason for CDKi discontinuation (binary)	
Progression	112 (80%)
Non-progression	28 (20%)
Time between CDKi discontinuation and post-CDKi therapy^†^	Median (IQR): 13 days (0-38)
Type of post-CDKi therapy^e^	
Chemotherapy	61 (44%)
Endocrine therapy	35 (25%)
Other targeted agent	30 (21%)
None	10 (7%)
CDKi	4 (3%)

^†^Information is not applicable for the 10 patients who did not receive any post-CDKi therapy.

^a^A patient with both visceral (eg, liver) and non-visceral (eg, bone) was categorized as “visceral.”

^b^Pre-CDKi chemotherapy drugs received by patients: capecitabine (*n* = 29), paclitaxel (*n* = 14), nab-paclitaxel (*n* = 10), doxorubicin (*n* = 8), and docetaxel (*n* = 7).

^c^If a patient received >1 endocrine therapy concurrently with a CDKi, the first endocrine therapy received is represented.

^d^Sequential CDKi regimens were combined if the regimen switch was not due to disease progression.

^e^“Post-CDKi therapy” was defined as the first cancer therapy the patient received after discontinuing CDKi. However, if a CDKi regimen was discontinued due to disease progression, this “Post-CDKi therapy” could include a different, subsequent CDKi. For patients who received drugs in >1 listed category, the following precedence rules were applied (from highest precedence to lowest): chemotherapy, CDKi or other targeted small molecule therapy, endocrine therapy. For example, a patient receiving any chemo, regardless of whether other drugs were taken, was placed in the chemotherapy group.

### Survival Following CDKi Discontinuation

**Figure 1.**
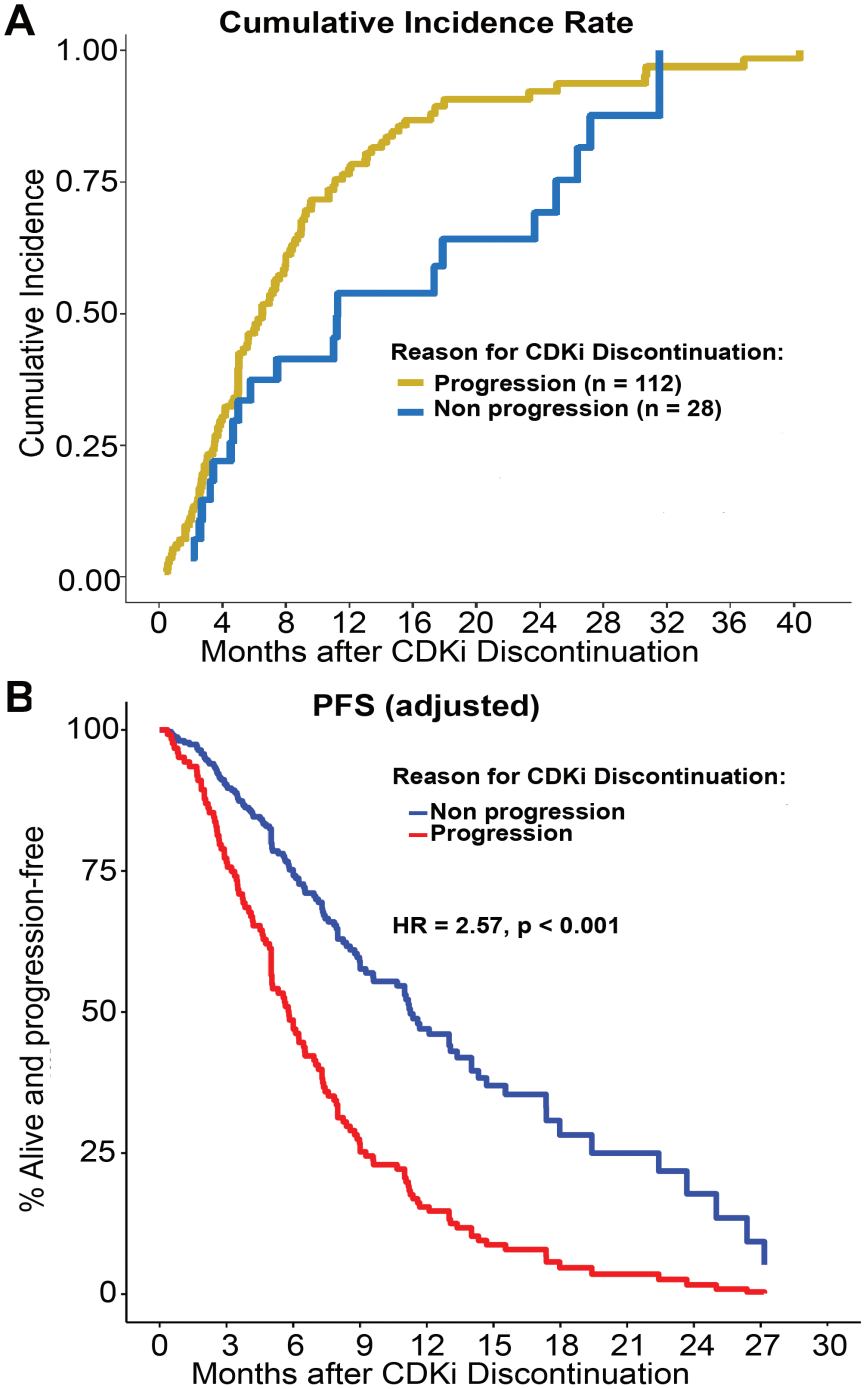
Progression post-CDKi (**A**) Cumulative incidence rate of post-CDKi progression or death stratified by reason for CDKi discontinuation. (**B**) PFS adjusted Cox model curve. Multivariable Cox proportional hazard regression adjusted for de novo metastases, visceral metastases, and duration of CDKi.

The median post-CDKi follow-up was 33 months. There were 97 on-study deaths (69% of patients), ranging from <1 to 40 months after CDKi discontinuation, and median overall survival (mOS) was 15.4 months (95% CI, 13.3-19.0). Median progression-free survival (mPFS) was 6.3 months (95% CI, 5.1-7.4). The mPFS for patients discontinuing CDKi because of progression (CDKi^prog^) was nearly half that of patients discontinuing CDKi for other reasons (CDKi^other^): 6.0 months (95% CI, 5.0-7.1) vs. 11.3 months (95% CI, 4.6-19.4), respectively; hazards ratio (HR) 2.53 (95% CI, 1.50-4.26), *P* = .001 (Table 2). The cumulative incidence rate of post-CDKi progression or death at 6 months for the CDKi^prog^ and CDKi^other^ groups was 51% (95% CI, 42%-61%) and 37% (95% CI, 22%-58%), respectively. At 12 months post-CDKi, rates were 86% (95% CI, 78%-91%) for CDKi^prog^ and 54% (95% CI, 36%-74%) for CDKi^other^ patients (Fig. 1A).

**Table 2.** Cox proportional hazards model evaluating progression-free survival after CDKi discontinuation.

Clinical characteristic	Median PFS (95% CI), months	HR (95% CI)	*P*-value
Age at CDKi discontinuation	N/A	0.99 (0.98-1.01)	.359
Age at CDKi discontinuation (binary)			
<65 years^	5.8 (5.0-7.9)	1.07 (0.75-1.52)	.709
≥65 years	7.0 (5.0-8.0)		
De novo metastatic breast cancer			
No^	6.9 (5.5-8.0)	1.33 (0.89-1.99)	.160
Yes	5.3 (3.8-7.0)		
Metastasis type (at time of metastatic diagnosis)			
Non-visceral^	7.3 (5.8-8.5)	1.35 (0.95-1.92)	.097
Visceral^†^	5.6 (4.6-7.4)		
Pre-CDKi therapy in metastatic setting			
No^	7.1 (5.8-8.0)	1.06 (0.73-1.54)	.767
Yes	5.6 (5.0-7.8)		
Pre-CDKi chemo in metastatic setting			
No^	7.1 (5.8-8.2)	1.36 (0.92-2.01)	.120
Yes	5.0 (3.8-6.1)		
Pre-CDKi anastrozole in metastatic setting			
No^	6.1 (5.0-7.3)	0.71 (0.44-1.15)	.162
Yes	8.8 (5.0-13.0)		
Pre-CDKi letrozole in metastatic setting			
No^	7.0 (5.8-8.5)	1.12 (0.78-1.61)	.539
Yes	5.0 (4.5-7.8)		
Pre-CDKi exemestane in metastatic setting			
No^	6.5 (5.0-7.4)	1.05 (0.69-1.61)	.817
Yes	6.2 (4.6-9.0)		
Pre-CDKi fulvestrant in metastatic setting			
No^	6.2 (5.0-7.3)	0.84 (0.57-1.23)	.362
Yes	7.6 (5.0-11.0)		
Pre-CDKi tamoxifen in metastatic setting			
No^	6.9 (5.6-7.9)	1.34 (0.75-2.40)	.316
Yes	5.0 (2.9-5.8)		
Pre-CDKi everolimus in metastatic setting			
No^	6.5 (5.3-7.6)	1.35 (0.78-2.37)	.287
Yes	5.1 (0.8-11.0)		
Pre-CDKi capecitabine in metastatic setting			
No^	6.9 (5.5-7.9)	1.30 (0.84-2.01)	.236
Yes	5.0 (3.2-8.0)		
Time between metastatic diagnosis and start of CDKi therapy, months	N/A	1.00 (0.99-1.00)	.912
Type of CDKi (first regimen)			
Palbociclib^	6.1 (5.0-7.4)		
Ribociclib	11.7 (6.5-NA)	0.73 (0.23-2.29)	.585
Abemaciclib	4.6 (2.2-NA)	0.91 (0.34-2.48)	.860
Endocrine therapy (specific) during CDKi^‡^			
Letrozole	6.3 (5.0-8.2)	Unreliable Cox model estimates due to small category counts	
Fulvestrant	6.1 (4.9-7.8)		
Anastrozole	10.7 (2.2-NA)		
Tamoxifen	7.9 (NA-NA); *n* = 1		
None	2.6 (NA-NA); *n* = 1		
Exemestane	N/A; *n* = 0		
Endocrine therapy (group) during CDKi^‡^			
NSAI^	6.5 (5.0-8.4)	1.36 (0.94-1.96)	.105
SERD	6.1 (4.9-7.8)		
Duration of CDKi^*^			
<12 months^	5.8 (4.8-6.5)	0.70 (0.48-1.03)	.073
≥12 months	8.0 (5.6-11.0)		
Reason for CDKi discontinuation			
Non-progression^	11.3 (4.6-19.4)	2.53 (1.50-4.26)	.001
Progression	6.0 (5.0-7.1)		
Type of post-CDKi therapy (TVC)^**^			
Chemotherapy^	6.1 (4.8-8.0)		
Endocrine tx	5.0 (4.1-11.0)	0.78 (0.49-1.22)	
CDKi	6.9 (5.1-NA)	0.83 (0.30-2.29)	
Other targeted tx	6.3 (3.5-7.9)	1.19 (0.75-1.87)	
None^	NA (3.7-NA)	0.54 (0.26-1.13)	

^Reference group for interpreting the hazard ratio (HR) for PFS. If the HR in a non-reference group row is >1, this indicates that this group has a greater risk of progression or death compared to the reference group. This elevated risk is statistically significant (at the nominal *α* level of .05) if its 95% CI is entirely above 1

^†^A patient with both visceral (eg, liver) and non-visceral (eg, bone) metastases was categorized as “visceral.”

^‡^If a patient received >1 endocrine therapy concurrently with a CDKi, the first endocrine therapy received is represented.

^*^Sequential CDKi regimens were combined if the regimen switch was not due to disease progression.

^**^“Post-CDKi therapy” was defined as the first cancer therapy the patient received after discontinuing CDKi. However, if a CDKi regimen was discontinued due to disease progression, this “Post-CDKi therapy” could include a different, subsequent CDKi. For patients who received drugs in >1 listed category, the following precedence rules were applied (from highest precedence to lowest): chemotherapy, CDKi or other targeted therapy, endocrine therapy.

Abbreviations: NSAI, non-steroidal aromatase inhibitor; SERD, selective estrogen receptor degrader (ie, fulvestrant); TVC, time-varying covariate (ie, predictor).

The following patient characteristics were associated with an elevated risk of progression or death after CDKi discontinuation (Table 2): de novo metastatic disease (HR 1.33 [95% CI, 0.89-1.99], *P* = .160), visceral disease at the time of metastatic diagnosis (HR 1.35 [95% CI, 0.95-1.92], *P* = .097), exposure to chemotherapy (HR 1.36 [95% CI:, 0.92-2.01], *P* = .120) or lack of exposure to anastrozole (HR 1.42 [95% CI, 0.88-2.30], *P* = .150) in the metastatic setting prior to CDKi, receipt of fulvestrant in combination with CDKi (HR 1.34 [95% CI, 0.93-1.93], *P* = .121), and CDKi duration <1 year (HR 1.42 [95% CI, 0.97-2.08], *P* = .073). After discontinuation of CDKi, mPFS estimates for next line therapies were: 5.0 months (95% CI, 4.1-11.0) for endocrine therapy, 6.1 months (95% CI, 4.8-8.0) for any chemotherapy, 6.3 months (95% CI, 3.5-7.9) for a non-CDKi targeted agent, and 6.9 months (95% CI, 5.1-NA) for a new CDKi. Nine of the 10 patients who did not receive any post-CDKi treatment died within 6 months after discontinuing CDKi.

In a multivariable analysis, the reason for CDKi discontinuation (progression vs. other) was the only significant predictor of PFS (HR 2.57 [95% CI, 1.53-4.33]; *P* < .001) (Fig. 1B). However, de novo metastatic disease (HR 1.33, *P* = .160), the presence of visceral metastases (HR 1.33, *P* = .119), and receipt of CDKi for less than 1 year (HR 1.43, *P* = .067) were also retained in the model. In a subgroup analysis of the 112 patients who discontinued CDKi because of disease progression, having visceral metastatic lesions was associated with shorter PFS (HR 1.64 [95% CI: 1.10-2.44], *P* = .015). The multivariable model for OS contained duration of CDKi and reason for CDKi discontinuation, with patients who ended therapy because of disease progression having a significantly higher risk of death (HR 2.49 [95% CI, 1.38-4.49], *P* = .002) than those who discontinued CDKi for another reason.

### Genomic Analyses

**Figure 2.**
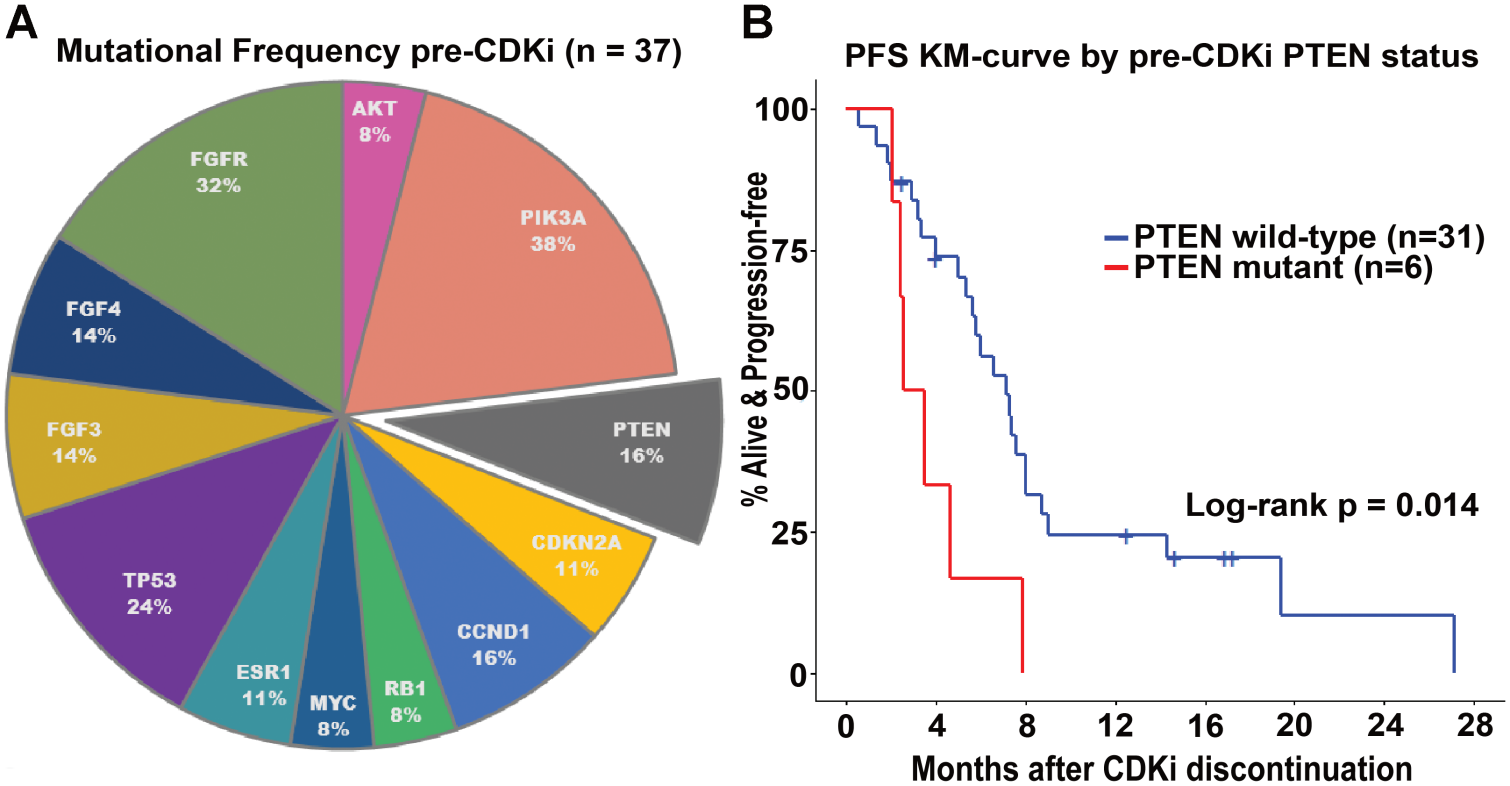
PTEN mutation as predictor of post-CDKi disease progression. (**A**) Frequency of mutations among subset of 37 patients with available clinical NGS prior to CDKi initiation. All but 4 of these patients had >1 mutation (of the 12 genes we analyzed). (**B**) Post-CDKi PFS Kaplan-Meier curves by pre-CDKi PTEN mutational status.

Clinical NGS was performed on pre-CDKi tumor samples of 37 patients in our cohort (5 primary site samples and 32 metastatic samples were sequenced). We evaluated the association between pre-CDKi mutation status and post-CDKi survival for 12 genes that had an alteration of any type in at least 2 of these 37 patients (Fig. 2A, Supplementary Table S1). *PTEN* alterations were present in 6 patients (16%) and included 4 copy loss, 1 splice site, and 1 frameshift mutation (Supplementary Table 2). *PTEN* had the strongest correlation with prognosis: those with a *PTEN* mutation had mPFS of 3 months and mOS of 4 months while wild-type patients had mPFS of 7 months (log-rank *P* = .014) and mOS of 18 months (log-rank *P* < .001) (Fig. 2B, Supplementary Table S1). In multivariable analyses, when adjusting for prognostic factors that had univariable model *P*-values < .20 and were retained after AIC-based backward elimination (pre-CDKi chemotherapy and pre-CDKi fulvestrant in the metastatic setting and ≥1 year of CDKi for PFS; pre-CDKi AKT mutational status and fulvestrant for OS), harboring a *PTEN* mutation increased the risk of progression and death (PFS HR 5.9 [95% CI, 1.9-18.3], *P* = .002; OS HR 17.3 [95% CI, 4.4-67.4], *P* < .001). Patients with *PTEN*-mutated tumors, compared to those with wild-type *PTEN*, were significantly more likely to experience rapid disease progression, when adjusting for pre-CDKi FGFR mutational status, chemotherapy in the metastatic setting, and age at CDKi discontinuation (odds ratio 88.3 [95% CI, 2.5-1.8 × 10^4^], *P* = .040).

## Discussion

Tumor progression following CDKi therapy is an unfortunate expectation for patients with HR+/HER2− MBC. In this single-institution study, 80% of patients experienced disease progression on CDKi therapy with a median PFS of 6.0 months that was significantly shorter than those who stopped CDKi therapy for reasons other than progression (11.3 months). In our analysis, visceral metastases at the time of metastatic diagnosis was a risk factor for progression or death, which is consistent with prior reports^27-34^ and suggestive of a more aggressive tumor biology underlying post-CDKi resistance.^35^ Furthermore, patients who progressed on a CDKi had a 51% chance of rapid disease progression, defined as progression or death within 6 months after CDKi discontinuation.

This phenomenon of rapid disease progression was first reported in a small case series of 4 patients who received CDKi in clinical trials, where post-CDKi survival varied from 4 to 25 months. A separate report found that post-CDKi treatment duration varied based on whether CDKi was given in the first or second line, with first-line CDKi associated with greater median time-to-treatment failure for the next line of therapy. Our analysis similarly revealed that prior treatment with chemotherapy was associated with greater risk for disease progression or death following CDKi discontinuation. Altogether, these findings expand upon existing efforts to understand the problem of post-CDKi disease progression in MBC by exploring predictors of progression and examining the reasons for CDKi discontinuation.

The optimal next-line therapy following CDKi is undetermined, yet there are observations that the time to next progression may vary with the type of therapy, with some studies reporting that patients treated with chemotherapy after receiving CDKi are significantly more likely to experience progressive disease.^23,27^ In our cohort, PFS following CDKi discontinuation was longest for the group of 4 patients whose next line of treatment was a different CDKi. Although based on a small sample size, this finding aligns with emerging data that suggest a role for continued CDKi therapy after progression.^36-40^ In a 1:1 randomized, prospective evaluation of 120 patients with HR+/HER2− MBC who had progressed on ET and CDKi therapy (with only 11% having prior ribociclib), those assigned to receive ribociclib with fulvestrant or exemestane had a significantly longer PFS (median 5.3 months; HR 0.56) than those receiving fulvestrant or exemestane alone (median 2.8 months).^41^ Similarly, recent studies report that 23%-37% of patients with HR+/HER2− MBC achieve durable responses greater than 6 months with abemaciclib therapy after progression on palbociclib or ribociclib.^36,38,39,42^ Additionally, evaluation of a separate retrospective cohort found that patients receiving abemaciclib after progression of disease on palbociclib had a median PFS of 5.3 months that was not meaningfully influenced by continued concurrent treatment with an ET.^38^ This suggests that continuation of CDKi (the same or alternative CDK targeting agent) at progression, similar to continuation of tyrosine kinase inhibitors in other malignancies at time of progression, may stave off tumor growth in some patients.^43-47^ Predicting which patients would benefit from such a strategy will require a better understanding of molecular changes occurring within a tumor and its microenvironment during treatment.

Our understanding of acquired CDKi resistance points to a complicated and heterogeneous landscape of mechanisms driving resistance, including adaptive changes that exploit redundancies in signaling pathways.^17,18,48-53^ Among these, constitutive PI3K/AKT/mTOR pathway activation (present in over 50% of HR+/HER2− MBC) plays a central role in tumorigenesis and can promote acquired endocrine and CDKi resistance.^38,54-60^ Emerging studies support a strategy for suppression of CDK4/6 activity combined with inhibition of PI3K/AKT/mTOR signaling to overcome CDKi resistance; however, no single gene alteration in the PI3K pathway has proven predictive of survival, suggesting that redundancies in the PI3K signaling network influence oncogenic potency.^6,37,38,50,51,54,55,58-61^ Mutational profiling available in a subset of our cohort revealed a preponderance of mutations in constituents of the FGFR/FGF and PI3K/AKT/mTOR pathways, which have known crosstalk mediating CDKi and endocrine resistance.^13,62^ Particularly, we observed that patients harboring a *PTEN* mutation before initiation of CDKi had significantly shorter post-CDKi PFS and 6 times the odds of experiencing rapid disease progression. Although a larger study is needed to confirm these findings, they align with preclinical studies showing that a loss of *PTEN* in breast tumor cells is sufficient to drive CDKi resistance.^51,59^ Upregulation of AKT activity stemming from *PTEN* loss promotes resistance to both CDKi and PI3Kα inhibitors, which is reversed by treatment with an AKT inhibitor (AKTi). Indeed, combining AKTi with CDKi and endocrine therapy in the preclinical setting was effective in reducing tumor growth and metastasis in cancers resistant to the combination of CDKi and endocrine therapy. The on-going CAPItello-292 trial (NCT04862663) is evaluating the AKTi, capivasertib, in combination with palbociclib and fulvestrant vs. palbociclib and fulvestrant in patients with HR+/HER2− MBC. Pending findings from this trial, it will be interesting to see if *PTEN* loss can serve as a biomarker to optimize upfront selection of patients that could benefit from this triplet regimen.

This study is limited by its relatively small sample size, the retrospective nature of the analysis, selection bias from physician choice of non-CDKi regimen, and pre-CDKi sequencing analyses that were performed on a limited subset of our cohort with available NGS data. Even so, this study, to our knowledge, provides the first real-world estimates of occurrence of disease progression following CDKi discontinuation among patients with HR+/HER2− MBC.

## Conclusion

Our findings suggest that a subset of progressing tumors are more aggressive upon discontinuation of CDKi therapy, adding to the clinical challenge imposed by lack of clinical practice guidelines for this unique population after progression. The widespread utilization of CDKi-based regimens in MBC underscores the need to understand how CDKi therapy alters tumor biology so that we can identify biomarkers of resistance or response, and optimize subsequent lines of treatment. Future studies molecularly characterizing patient tumors before and after CDKi are needed to help design clinical trials for patients with HR+/HER2− MBC.

## Supplementary Material

oyad035_suppl_Supplementary_Table_S1

oyad035_suppl_Supplementary_Table_S2

## Data Availability

The data that support the findings are not publicly available, but may be made available upon reasonable request from the corresponding author, Dr Zahi Mitri.
