# Supplementary material for: Real-World Evaluation of Disease Progression After CDK 4/6 Inhibitor Therapy in Patients With Hormone Receptor-Positive Metastatic Breast Cancer
Source: Oncologist. 2023 Mar 22;28(8):682–90. doi: 10.1093/oncolo/oyad035 (PMC10400146; doi:10.1093/oncolo/oyad035)
Supplement: oyad035_suppl_Supplementary_Table_S1 [file oyad035_suppl_supplementary_table_s1.docx]

**Supplemental Table 1. Cox proportional hazards model evaluating progression-free survival and overall survival by pre-CDKi gene mutation.**

|  | | | **PFS** | | **OS** | |  |
| --- | --- | --- | --- | --- | --- | --- | --- |
| **Gene** | **Pre-CDKi call** | **n (%)** | **Median (95% CI), months** | **Cox adj. HR; p-value^†^** | **Median (95% CI), months** | **Cox adj. HR; p-value^†^** | |
| AKT | wild-type^  mutated | 34 (92%)  3 (8%) | 6.5 (4.6 - 7.8)  3.2 (2.1 - NA) | 1.69; 0.449 | 15.8 (8.4 – 23.3)  3.2 (2.4 – NA) | 6.02; 0.070 | |
| CCND1 | wild-type^  mutated | 31 (84%)  6 (16%) | 5.3 (3.4 - 7.4)  7.8 (6.5 - NA) | PH fail | 16.6 (8.0 – 23.3)  14.9 (7.0 – NA) | 0.67; 0.470 | |
| CDKN2A | wild-type^  mutated | 33 (89%)  4 (11%) | 5.8 (4.0 - 7.6)  7.5 (1.3 - NA) | 1.96; 0.264 | 16.6 (7.6 – 23.3)  15.8 (9.4 – NA) | 0.43; 0.280 | |
| ESR1 | wild-type^  mutated | 32 (86%)  5 (14%) | 6.5 (3.5 - 7.8)  5.8 (0.5 - NA) | 0.68; 0.506 | 13.9 (8.0 – 22.5)  25.1 (0.5 – NA) | 0.45; 0.289 | |
| FGF3* | wild-type^  mutated | 30 (81%)  5 (14%) | 5.6 (3.4 - 8.0)  7.6 (6.5 - NA) | PH fail | 16.6 (8.4 – 23.3)  13.9 (7.0 – NA) | 0.81; 0.713 | |
| FGF4* | wild-type^  mutated | 30 (81%)  5 (14%) | 5.6 (3.4 - 8.0)  7.6 (6.5 - NA) | PH fail | 16.6 (8.4 – 23.3)  13.9 (7.0 – NA) | 0.81; 0.713 | |
| FGFR | wild-type^  mutated | 26 (70%)  11 (30%) | 7.3 (5.0 – 14.3)  6.3 (2.5 – NA) | 1.41; 0.484 | 16.6 (8.4 – 29.9)  13.9 (3.5 – 23.3) | 0.73; 0.513 | |
| MYC | wild-type^  mutated | 35 (95%)  2 (5%) | 6.5 (5.0 – 8.0)  3.4 (2.1 – NA) | 0.53; 0.561 | 15.8 (8.4 – 23.3)  2.4 (2.4 – NA) | 2.00; 0.525 | |
| PIK3CA | wild-type^  mutated | 22 (59%)  15 (41%) | 5.8 (5.0 – 12.1)  7.4 (4.0 – NA) | 1.56; 0.372 | 16.6 (8.0 – 23.3)  10.7 (3.5 – NA) | 1.11; 0.814 | |
| **PTEN** | **wild-type^**  **mutated** | **31 (84%)**  **6 (16%)** | **7.1 (5.3 - 8.0)**  **3.0 (2.1 - NA)** | **5.91; 0.002** | **17.9 (13.3 – 29.9)**  **3.9 (2.4 – NA)** | **7.74; <0.001** | |
| RB1 | wild-type^  mutated | 33 (89%)  4 (11%) | 6.5 (5.0 – 8.0)  6.0 (2.1 – NA) | 0.38; 0.201 | 13.9 (8.0 – 22.5)  23.3 (2.4 – NA) | 0.36; 0.184 | |
| TP53 | wild-type^  mutated | 28 (76%)  9 (24%) | 7.1 (5.0 – 12.1)  5.8 (3.4 – NA) | 1.16; 0.755 | 15.8 (8.0 – 31.5)  16.6 (0.5 – 23.3) | 1.51; 0.364 | |
| Genes included in the table are those for which at least 2 patients had a mutation of any type, according to next-generation sequencing of a sample collected prior to the patient starting a CDKi regimen.  ^†^ The adjusted Hazard Ratios (HR) and Wald p-values are from Cox models that included the following covariates selected with AIC-based backward elimination. For PFS: chemotherapy exposure prior to CDKi in the metastatic setting, pre-CDKi fulvestrant in the metastatic setting, duration of CDKi (< 1 year vs. ≥ 1 year). For OS: receipt of pre-CDKi anastrozole in the metastatic setting, duration of CDKi.  ^ Reference group for interpreting the Hazard Ratios  PH fail = the Cox regression model assumption of proportional hazards was not defensible so no Cox output is provided.  *The output for FGF3 and FGF4 is identical since the same 5 patients had mutations in these two genes. | | | | | | | |
