# Supplementary material for: Real-World Evaluation of Disease Progression After CDK 4/6 Inhibitor Therapy in Patients With Hormone Receptor-Positive Metastatic Breast Cancer
Source: Oncologist. 2023 Mar 22;28(8):682–90. doi: 10.1093/oncolo/oyad035 (PMC10400146; doi:10.1093/oncolo/oyad035)
Supplement: oyad035_suppl_Supplementary_Table_S2 [file oyad035_suppl_supplementary_table_s2.docx]

**Supplemental Table 2. Metastatic disease treatment details and post-CDKi PFS for the 6 patients with a pre-CDKi PTEN mutation detected by NGS.**

| **ID** | **PTEN mutant type** | **Pre-CDKi chemo in met. setting** | **CDKi line number in met. setting** | **CDKi duration (months)** | **Reason for CDKi discont.** | **PFS time (months)^†^** |
| --- | --- | --- | --- | --- | --- | --- |
| 1 | copy loss | yes | 6 | 19.1 | progression | 2.1 |
| 2 | copy loss | yes | 2 | 8.2 | progression | 2.4 |
| 3 | splice site | no | 2 | 6.3 | progression | 2.5 |
| 4 | copy loss | no | 1 | 18.0 | progression | 3.5 |
| 5 | T319fs*6 | yes | 5 | 5.7 | progression | 4.6 |
| 6 | copy loss | no | 3 | 8.6 | progression | 7.8 |
| ^†^ Progression-free survival time measured from CDKi discontinuation. All 6 patients had a PFS event. | | | | | | |
